# Triterpenic Acid Amides as Potential Inhibitors of the SARS-CoV-2 Main Protease

**DOI:** 10.3390/molecules28010303

**Published:** 2022-12-30

**Authors:** Dmitry S. Baev, Mikhail E. Blokhin, Varvara Yu. Chirkova, Svetlana V. Belenkaya, Olga A. Luzina, Olga I. Yarovaya, Nariman F. Salakhutdinov, Dmitry N. Shcherbakov

**Affiliations:** 1 N.N. Vorozhtsov Novosibirsk Institute of Organic Chemistry, 9 Lavrent’iev Avenue, 630090 Novosibirsk, Russia; 2 Department of Physical-Chemistry Biology and Biotechnology, Altay State University, 61 Lenina Avenue, 656049 Barnaul, Russia; 3 State Research Center of Virology and Biotechnology VECTOR, Rospotrebnadzor, 630559 Koltsovo, Russia

**Keywords:** triterpene amide, SARS-CoV-2, main protease, molecular modeling, FRET, antiviral

## Abstract

Although the incidence and mortality of SARS-CoV-2 infection has been declining during the pandemic, the problem related to designing novel antiviral drugs that could effectively resist viruses in the future remains relevant. As part of our continued search for chemical compounds that are capable of exerting an antiviral effect against the SARS-CoV-2 virus, we studied the ability of triterpenic acid amides to inhibit the SARS-CoV-2 main protease. Molecular modeling suggested that the compounds are able to bind to the active site of the main protease via non-covalent interactions. The FRET-based enzyme assay was used to reveal that compounds **1e** and **1b** can inhibit the SARS-CoV-2 main protease at micromolar concentrations.

## 1. Introduction

Searching for chemical compounds that are capable of exerting an antiviral effect against the SARS-CoV-2 virus by inhibiting its main protease (Mpro), a key enzyme of the virus life cycle, continues to be a relevant trend in drug design [1]. The main protease cleaves the translated viral polyprotein 1AB at 11 specific sites. The site recognition sequence in most cases consists of the chain segment (Leu-Gln)-(Ser-Ala-Gly); the bond between glutamine and serine is cleaved. Inhibition of the main protease stops viral replication. No proteases possessing the same cleavage specificity are known among human enzymes, which may indicate the absence of toxicity of potential inhibitors of the SARS-CoV-2 Mpro [2,3].

Many natural triterpenoids have an antiviral effect [4]. Thus, glycyrrhizic acid may exert an antiviral activity against coronaviruses [5,6]. Betulinic acid derivatives exhibit activity against HIV, the coronavirus, and the respiratory syncytial virus [7]. Triterpenoids of the ursane series also exhibit antiviral activity against HIV, the rotavirus, and the influenza virus [8]. Oleanolic [9] and ursolic acids [10], as well as their derivatives, can inhibit HIV proteases. In addition, oleanolic acid and its derivatives inhibit HCV protease [11]. Betulonic acid amides have shown antiviral activity against a wide range of viruses [12]. The presence of an amide bond has also been shown to increase activity against HIV [13]. A literature review revealed that positions 3 and 28 of the triterpene backbone play an important role in triterpenoid activity. Their chemical modification may allow one to produce new drugs that have better activity and fewer side effects.

We are considering the possibility of an inhibitory effect on the SARS-CoV-2 Mpro of triterpenoid derivatives that have previously been synthesized in our laboratory as potential dual agonists of PPAR-α,γ [14]. The triterpene amides tested in this study for protease affinity contain a large pharmacophore fragment, (*S*)-2-ethoxy-3-(4-hydroxyphenyl) propanoic acid in the amide portion, which is characteristic of glitazars, compounds which are used for treating metabolic syndrome. In order to reveal the effect of this fragment on protease affinity, we synthesized a new compound containing a *p*-menthane (monoterpene) fragment instead of the triterpene fragment as a pharmacophore of glitazars.

The active site of the SARS-CoV-2 Mpro contains catalytic amino acids Cys145 and His41, and resides between the N-terminal domains I and II of the monomer of this enzyme [15]. Most studies focusing on potential inhibitors were devoted to molecules that could covalently bind to the SH group of the catalytic amino acid residue Cys145 of the protease. The mechanisms of such covalent binding were in most cases represented by either Michael addition or nucleophilic addition to a double or triple bond. Less attention has been paid to potential non-covalent inhibitors. In the case of non-covalent inhibition, reversible competitive substitution of the enzyme’s active site takes place, preventing its interaction with the substrate. Molecular docking was used to study the theoretical possibility that triterpene amides can block the Mpro active site due to non-covalent interactions. The compounds were tested for their ability to inhibit the major protease via the FRET-based enzyme assay.

## 2. Results

### 2.1. Molecular Modeling

**Figure 1:**
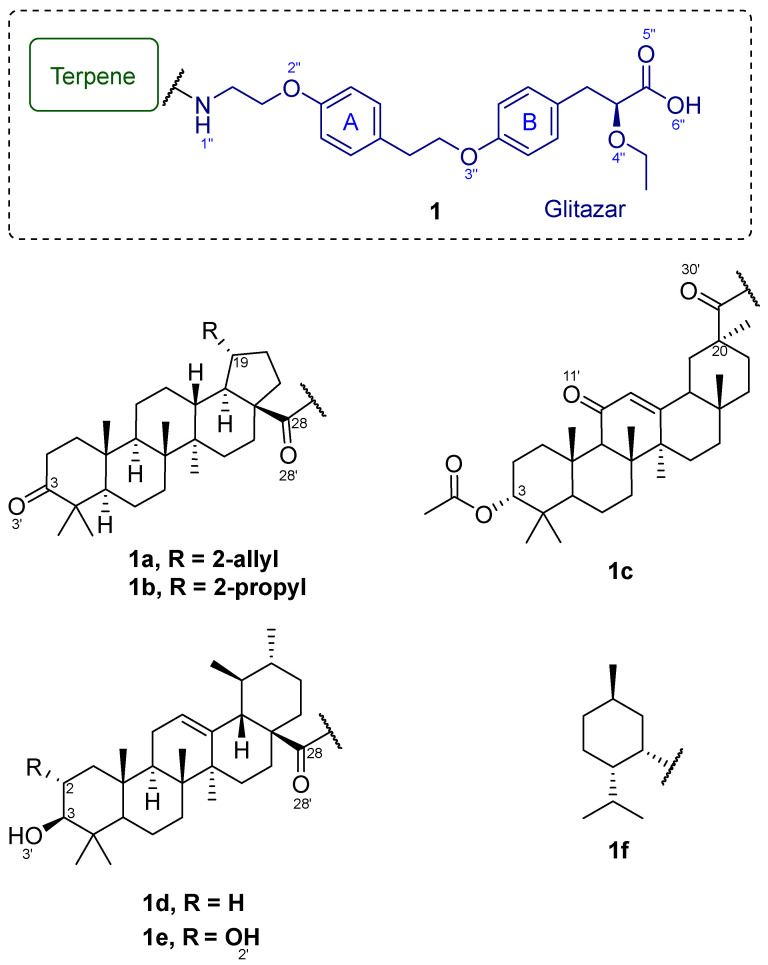
Triterpene amides (**1a–e**) and neomenthylamine derivative (**1f**).

Among the triterpene amides, compounds **1a–e** shown in Figure 1 exhibited the highest theoretical affinity in molecular modeling. Neomenthylamine derivative **1f** did not exhibit high theoretical affinity in molecular modeling, and was chosen as a negative control.

**Figure 2:**
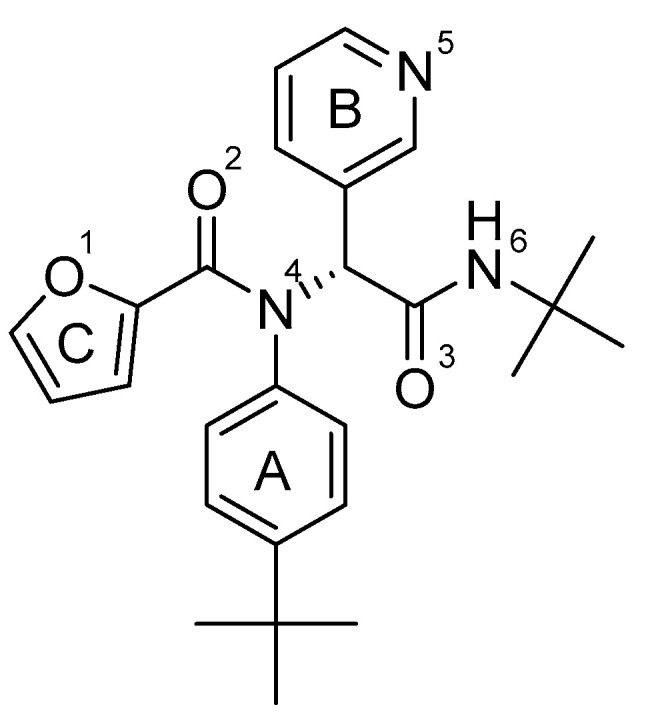
Non-covalent SARS-CoV-2 Mpro inhibitor ML188.

The estimated energy of interaction (the docking score) between most conjugates and the active site of the SARS-CoV-2 Mpro was significantly lower or comparable to that of the ML188 model inhibitor (Figure 2). The IFD score parameter lay within a narrow range for all of the compounds, and was comparable to that of ML188. Apparently, the compounds caused changes in the positions of amino acid side chains and their rotations, similarly to those caused by the model inhibitor ML188. Considering the Emodel energy parameter, one can see that compounds **1a** and **1c** had the lowest energies, which indicates a greater number of non-covalent interactions between these molecules and the active site of the main protease, although their docking parameter score was higher than that of compound **1e** (Table 1).

**Figure 3:**
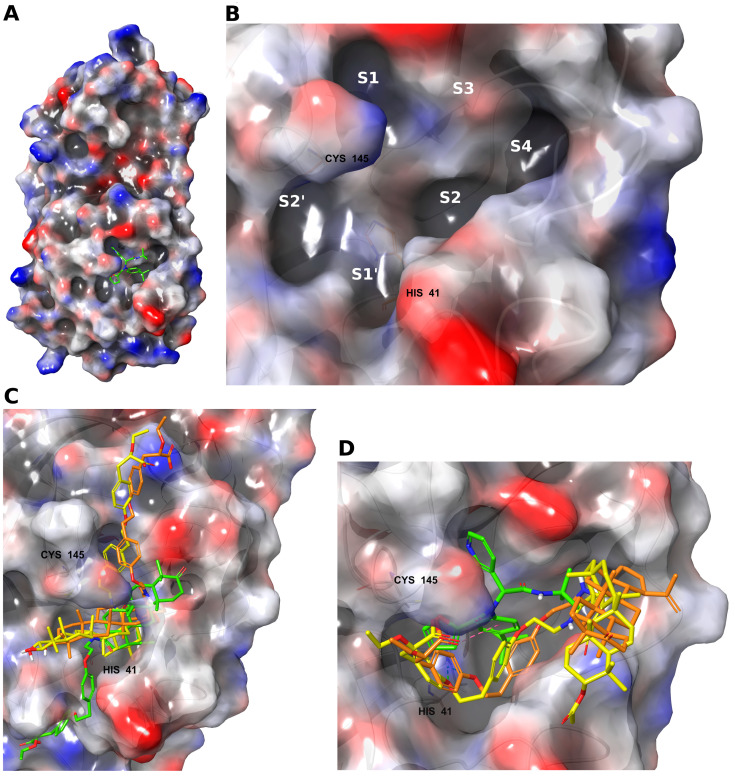
(**A**) The SARS-CoV-2 main protease subunit with an inhibitor at the active site. (**B**) Active site subpockets. The superpositions of compounds in the active site of the SARS-CoV-2 main protease show differences in their location with respect to the active site: (**C**) 1e (yellow), 1d (orange), and 1b (green); (**D**) 1c (yellow), 1a (orange), and ML188 (green).

**Figure 4:**
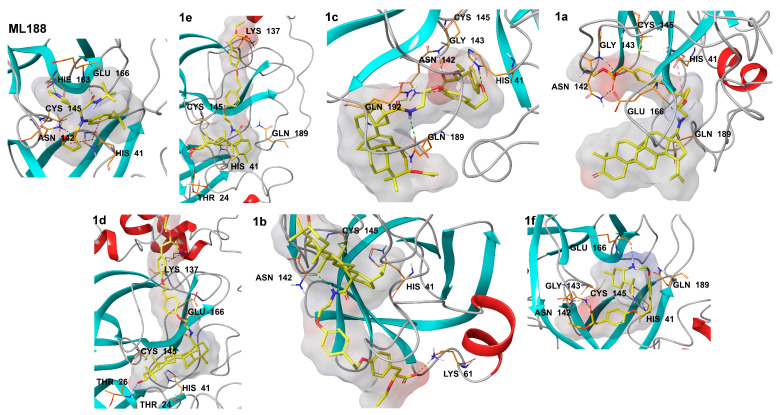
Non-covalent interactions of compounds (**1a**–**f**) in the active site of the SARS-CoV-2 Mpro compared to the inhibitor ML188 (ML188). Non-covalent interactions are shown by dotted lines: green—hydrogen bonds, orange—ionic interactions, magenta—stacking interactions, dark green—pi-cation interactions.

Apparently, the key factor of the inhibitory effect on the SARS-CoV-2 Mpro was the ability of the ligand to bind the amino acid residues of the flexible loop region 141–145, which formed the oxyanion hole of the enzyme [4]. The oxyanion hole is a pocket in the enzyme’s active site that stabilized the negative charge of the transition state on deprotonated oxygen or alkoxide. Transition state stabilization lowered the activation energy required for the reaction, and thus promoted catalysis [16]. The catalytic center of the SARS-CoV-2 Mpro (Figure 3A) consists of two pockets: S1’, where interaction with His41 occurs, and S2’, where the catalytic amino acid residue Cys145 is located. In addition, studies have shown that the active site of the main protease is complemented by pockets that contribute to stabilization of the inhibitor near the enzyme’s catalytic site. In the S1 pocket, inhibitors can interact with amino acids Glu166 and Phe140; in the S2 pocket, with His41, Hie163, and His164; in the S3 pocket, with Gln189 and Met49; and in the S4 pocket, with Pro168 [3,5,17] (Figure 3B). Due to its branched structure, the model inhibitor ML188 effectively interacted with the amino acids of the catalytic center of the Mpro (here and below, the non-covalent interactions are illustrated in Figure 4 and Figure S7), and penetrated into the stabilizing pockets of the active site (Figure 3D). The aromatic rings in its structure allowed it to interact with the pi system of the His41 catalytic amino acid.

Triterpenic amides can be oriented in the active site of the SARS-CoV-2 Mpro in various ways. (S)-2-Ethoxy-3-(4-hydroxyphenyl) propanoic acid residue and the triterpene backbone were located both near the catalytic center of the enzyme and outside it. Despite the lowest docking score, compound **1e** interacted exclusively with Gln189. Apparently, the low interaction energy in this case was achieved due to the placement of the (S)-2-ethoxy-3-(4-hydroxyphenyl) propanoic acid moiety in the groove between domains I and II of the Mpro subunits. Compound **1d** occupied a position similar to that of compound **1e**: its triterpene backbone was located near the active site of the Mpro. Compound **1c** also had a triterpene backbone near the catalytic center, and the (S)-2-ethoxy-3-(4-hydroxyphenyl) propanoic acid fragment occupied various positions outside the active site of the Mpro (Figure 3C). Compounds **1a** and **1c** demonstrated a more favorable position near the active site of the Mpro, confirming the lowest Emodel energies (Figure 3D). They could interact with amino acids in the chain region 141–145 (hydrogen bonds) and with the catalytic amino acid His41 (stacking interaction), due to the (S)-2-ethoxy-3-(4-hydroxyphenyl) propanoic acid residue located near the active site of the main protease, as well as with the stabilizing amino acids Glu166 and Gln189 using the polar groups of the triterpene backbone. Compound **1f**, due to the absence of triterpenoid structures, could theoretically interact with the amino acids of the active site of the main protease due to the polar groups and aromatic cycles of the (S)-2-ethoxy-3-(4-hydroxyphenyl) propanoic acid fragment. Features and distances of non-covalent interactions of the new triterpene amides with amino acids of the SARS-CoV-2 Mpro active site are shown in Table 2.

### 2.2. Chemistry

**Scheme 1:**
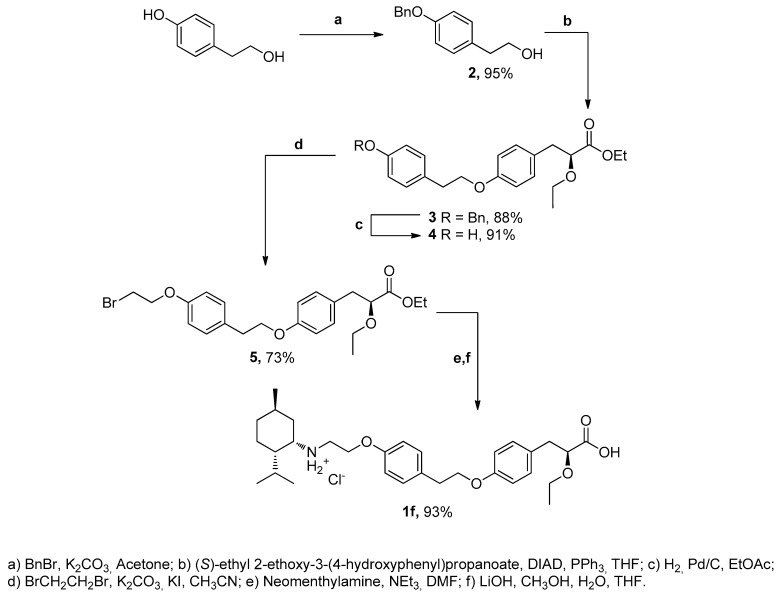
The synthetic route for the synthesis of compound **1f**.

Neomenthylamine derivative **1f** was synthesized in five steps (Scheme 1). At the first step, compound **2** was obtained with a 95% yield, using an adapted procedure of phenol benzylation. The Mitsunobu reaction between alcohol **2** and (*S*)-ethyl 2-ethoxy-3-(4-hydroxyphenyl) propanoate in the presence of diisopropylazodicarboxylate (DIAD) and triphenylphosphine in THF gave rise to ester **3**. Debenzylation of **3** in the presence of H_2_ and 10% Pd/C in ethyl acetate was carried out with a 91% yield. Phenol **4** further reacted with dibromoethane in excess K_2_CO_3_ in acetonitrile to give rise to bromide **5** with a 73% yield. The interaction between bromide **5** and neomenthylamine followed by hydrolysis of the ether group gave rise to amine **1f** as a hydrochloride salt with a 93% yield.

**Scheme 2:**
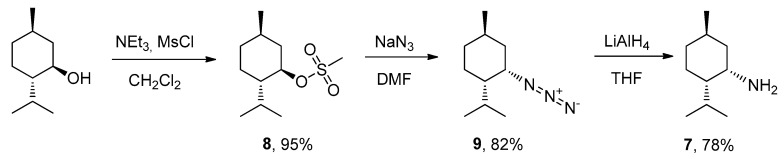
Synthesis of neomenthylamine.

The synthesis of neomethylamine **7** was carried out in three steps: mesylation of (–) -menthol, replacement of the mesyl group by an azide group, and its subsequent reduction to an amino group (Scheme 2).

Mesylate **8** was obtained via the reaction between (1R)-(**–**)-menthol and mesyl chloride, in the presence of NEt_3_ in methylene chloride for 24 hrs, with a 95% yield. Next, mesylate **8** was reactivated with sodium azide in DMF, resulting in formation of azide **9** isolated with an 82% yield. The conformation was reversed from the R to the S configuration. Amine **7** was prepared by the reduction of azide **9** with an excess of LiAlH_4_ in anhydrous THF. The purification of neomethiamine **7** was performed with acid-base extraction.

### 2.3. SARS-CoV-2 Mpro Inhibiting Activity

Previously, our research team developed a FRET-based enzyme assay to search for SARS-CoV-2 Mpro inhibitors. Effective protease inhibitors were detected using this surrogate test system [18]. In addition, this surrogate model enabled us to elucidate the mechanism of antiviral action of effective SARS-CoV-2 inhibitors, based on natural usnic acid [19]. The inhibitory activity of the synthesized conjugates against the SARS-CoV-2 Mpro was studied in vitro using the previously developed FRET-based enzyme assay (Table 3). Compounds **1e** and **1c** showed significant inhibitory activity. Conjugates **1a**, **1d**, and **1b** were less active, but also exhibited a significant effect. Compound **1f** confirmed its role as a negative control, and did not exert antiprotease activity in the in vitro experiment.

## 3. Discussion

The triterpenic acids selected in this study are known for their antiviral properties. Thus, certain lupane-type triterpenoids, such as betulonic and dihydrobetulonic acids, were shown to be potential inhibitors of the SARS-CoV Mpro. In contrast, ursolic triterpenoids such as ursolic and corosolic acids exhibited a more pronounced activity compared to that of lupane-type triterpenoids [20] because the presence of the hydroxyl group improves activity, while the keto group reduces the activity. In addition, glycyrrhetic acid and its derivatives (its glycosides in particular) are recognized as potential SARS-CoV-2 inhibitors [21].

**Figure 5:**
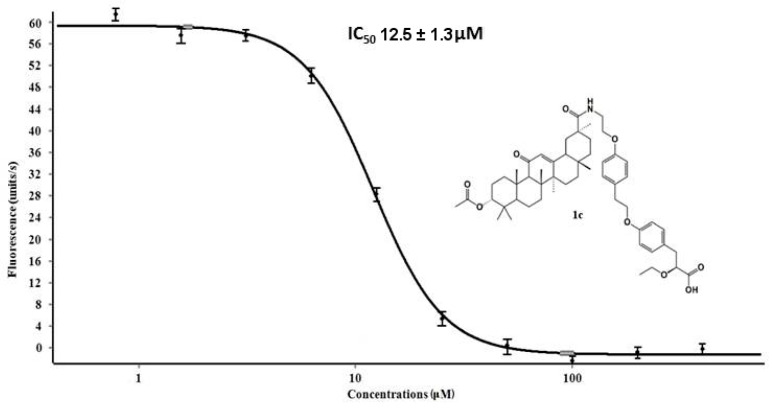
The dose–response curve and IC_50_ value of conjugate **1c** against the SARS-CoV-2 MPro.

Considering the relationship between the structure and activity of compounds, all triterpenoid amides at position 28 of the triterpene backbone with an (S)-2-ethoxy-3-(4-hydroxyphenyl) propanoic acid substituent (**1e**, **1a, 1d, 1b**) and an amide at position 20 of the triterpene backbone with the same substituent and an acetyl group at position 3 (**1c**) exhibited in vitro antiprotease activity. The interaction between triterpene amides and the SARS-CoV-2 Mpro was found to be more efficient if the (S)-2-ethoxy-3-(4-hydroxyphenyl) propanoic acid fragment was located near the enzyme’s active site, which could form hydrogen bonds and stacking interactions with the amino acids of the catalytic center due to the presence of aromatic cycles and polar groups. The formation of the amide moiety **1c** at position 20 of the triterpene backbone that is characteristic of glycyrrhetic acid provided additional advantages in binding to the active site of the Mpro, which manifested itself in the lowest IC_50_ value for this compound (Figure 5). According to the molecular modeling data, the presence of an acetyl group at position 3 of the triterpene backbone of **3c** did not have a significant effect on binding. The presence of a hydroxyl group at position 2 of the triterpene backbone of corosolic acid amide **1e** increased its Mpro inhibitory activity four-fold, compared to ursolic acid amide **1d**. This trend correlated well with the molecular modeling data, where compound **1e** showed a significantly lower docking score compared to compound **1d**. Hydrogenation of the double bond in the 2-allyl substituent at position 19 of the triterpene backbone of betulonic acid amide **1a** did not alter the antiprotease activity of dihydrobetulonic acid amide **1b**.

Compound **1f** did not show antiprotease activity in vitro, although it could theoretically interact with key amino acids of the active site of the main protease. Apparently, the absence of a triterpene backbone in this compound led to instability of interaction with the enzyme surface, due to a reduced number of hydrophobic interactions.

## 4. Materials and Methods

### 4.1. Molecular Modeling

All theoretical calculations were carried out using the Schrodinger Small Molecule Drug Discovery Suite 2020-2 software package [22]. The geometric parameters of the proteins were downloaded from the Protein Data Bank [23]. The XRD model of the SARS-CoV-2 main protease with a non-covalent inhibitor ML188 [24] (PDB ID 7L0D) was chosen for molecular modeling. The structure of the model protein was prepared by adding and minimizing hydrogen atoms, adding missing amino acid side chains, restoring bond multiplicities, removing solvent molecules, and optimizing the structures in the OPLS4 force field [25]. The geometric parameters of the ligands were optimized using the OPLS4 force field, taking into account all possible conformations. Molecular docking was performed using the IFD [26] (induced fit docking) protocol, which employs the Glide [27] and Prime [28] programs to predict the ligand positions in the binding site, taking into account their influence on the enzyme structure. The following conditions were applied: flexible protein and ligands; docking area size of 20 Å; amino acids within 5 Å of the ligand were taken into account to optimize its effect. Docking results were ranked by evaluating the following calculated parameters: docking score (based on GlideScore with the exception of penalties that take into account energy parameters that negatively affected binding); ligand efficiency (LE, where the distribution of the estimated energy over the heavy atoms of the ligand was considered); the model energy value parameter (Emodel, including the GlideScore value, the energy of non-covalent interactions, and the energy spent on the formation of stacking interactions of compounds in the binding site); and the IFD parameter (IFDscore, including the GlideScore value and the change in protein free energy, indicating the result of structural changes in the target). The docking algorithm was validated by redocking the standard inhibitor ML188, followed by the calculation of the RMSD between the coordinates of the co-crystallized molecule and the best docking pose. The RMSD value was 1.411 Å.

### 4.2. Chemistry

The ^1^H and ^13^C NMR spectra (Figures S1–S6) of the compounds in CDCl_3_ solutions were measured in a Bruker AV-400 spectrometer (400.13 and 100.61 MHz, respectively). The residual signals of the solvent were used as references (δH 7.27, δC 77.1 ppm). Chemical shift measurements were calculated in ppm; the coupling constants (J) were calculated in Hertz (Hz). Column chromatography employed Merck silica gel (63–200 µm). Thin-layer chromatography was performed using TLC Silica gel 60F254, Merck (Darmstadt, Germany).

All of the chemicals were used as received, unless otherwise noted. Reagent-grade solvents were redistilled prior to use. Synthetic starting materials, reagents, and solvents were purchased from Acros Organics (Geel, Belgium), Sigma-Aldrich (St. Louis, MO, USA), and AlfaAesar (Heysham, UK). Ursolic, betulonic, and dihydrobetulonic acids were purchased from the Departmental Pilot of the Novosibirsk Institute of Organic Chemistry, SB RAS. Acetylglycyrrhetinic acid was donated by colleagues from the Department of Medicine at the Novosibirsk Institute of Organic Chemistry, SB RAS. Corosolic acid was synthesized from ursolic acid according to the procedure described in [29]. The obtained spectral data coincided with those found in the literature.

The triterpenic acid amides containing (S)-ethyl 2-ethoxy-3-(4-hydroxyphenyl) propanoic acid moiety were synthesized according to the method described earlier [13].

2-(4-(benzyloxy)-phenyl) ethanol (**2**)

Tyrosol (0.12 mol) was dissolved in 100 mL of acetone; then, 0.19 mol of calcined potassium carbonate previously ground in a mortar was added and left to stir for 30 min. Benzyl bromide (0.13 mol) was then added, drop by drop, and the mixture was left to boil for 8 h. The reaction was monitored by TLC in a 4:1 hexane:ethyl acetate system. After the reaction completed, the reaction mixture was poured into 700 mL of water and left to stir for 30 min. The precipitate was filtered off and left to dry. Purification was performed via precipitation from a hexane–ethyl acetate mixture.

The material obtained was pearl-colored powder, 26.00 g, with a yield of 95%. The spectral data corresponded to data in the literature [17].

(S)-ethyl 3-(4-(4-(4-(benzyloxy)-phenethoxy)-phenyl)-2-ethoxypropanoate (**3**)

(S)-ethyl 2-ethoxy-3-(4-hydroxyphenyl)-propanoate (23 mmol) and 24 mmol of PPh_3_ were mixed in a 250 mL round-bottom flask containing 22 mmol of 2-(4-(benzyloxy)-phenyl) ethanol, and dissolved in 70 mL of THF. The reaction mixture was cooled in an ice bath; then, 23 mmol of DIAD in an argon current was added and left to stir for 24 h. After the reaction completed, the solvent was evaporated; purification was performed via column chromatography in a 4:1 hexane:ethyl acetate system.

The material obtained= was a yellow oil, 8.67 g, with a yield of 88%. The spectral data corresponded to that in the literature [30].

(S)-ethyl-2-ethoxy-3-(4-(4-hydroxyphenethoxy)-phenyl) propanoate (**4**)

(S)-ethyl-3-(4-(4-(4-(benzyloxy)-phenethoxy)-phenyl)-2-ethoxypropanoate (1.03 g) was dissolved in 25 mL of methanol; the solution was degassed; then, 103 mg of 10% palladium on carbon was added, purged with hydrogen, and left in a hydrogen atmosphere under stirring for 24 h. The reaction was monitored by TLC (eluent: 20:1 CHCl_3_:MeOH). The catalyst was filtered off, and the solvent was evaporated. The product was used without further purification.

The material obtained was white crystals, 0.782 g, with a yield of 95%. The spectral data corresponded to data in the literature [17].

Ethyl 3-(4-(4-(4-(2-bromoethoxy)-phenethoxy)-phenyl)-2-ethoxy propanoate (**5**)

(S)-ethyl 2-ethoxy-3-(4-(4-hydroxyphenethoxy)-phenyl) propanoate (0.83 mmol), 4.2 mmol of 1,2-dibromoethane, 2.5 mmol of potassium carbonate, and potassium iodide crystals were placed into a 50 mL flask containing 30 mL of acetonitrile, purged with argon, and left to stir for 3 days; the reaction was controlled by TLC in a 7:1 hexane:ethyl acetate system. Potassium carbonate was then filtered off, the solvent was evaporated, and the product was purified with column chromatography in a 6:1 hexane:ethyl acetate system.

The material obtained was a yellow oil, 0.261 g, with a yield of 68%. ^1^H-NMR: 1.17 (3 H, t, J = 7.0), 1.23 (3 H, m), 2.95 (2 H, m), 3.04 (2 H, m), 3.30–3.40 (1 H, m), 3.54–3.67 (3 H, m), 3.97–4.21 (5 H, m), 4.28 (2 H, t, J = 6), 6.82–6.88 (4 H, m), 7.15–7.21 (4 H, m). ^13^C-NMR: 14.2, 15.0, 29.3, 34.8, 38.4, 60.8, 66.1, 67.8, 68.7, 80.3, 114.2 (3C), 114.7, 130.0, 130.3 (2C), 130.4 (3C), 157.2, 157.6, 172.5. Found: m/z 464.1198 [M]+. C_26_H_37_NO_5_. Calculated: M 464.1198.

(S)-ethyl 2-ethoxy-3-(4-(4-(2-(mentylamino)-ethoxy)-phenethoxy)-phenyl) propanoate (**6**)

(S)-ethyl-3-(4-(4-(2-bromoethoxy) phenethoxy)-phenyl)-2-ethoxy propanoate (2.1 mmol) and 6.3 mmol of the corresponding amine in 12 mL of DMF were placed into a 50 mL round-bottom flask; the solution was cooled, and 3.2 mmol of NEt_3_ was then added dropwise. The reaction mixture was purged with argon and left to stir for 3 days; the reaction was controlled by TLC (eluent, 4:1 hexane:ethyl acetate). After the reaction completed, the mixture was poured into 70 mL of water; the aqueous layer was extracted with ethyl acetate; the organic layer was washed with saturated NaCl solution and dried over magnesium sulfate. The solvent was evaporated on a rotary evaporator; the product was purified with column chromatography in a 100:1 CHCl_3_:MeOH system.

The material obtained was an orange oil, 0.37 g, with a yield of 72%. ^1^H-NMR: 0.74–0.97 (13 H, m), 1.16 (8 H, m), 1.35–1.78 (6 H, m), 1.89 (1 H, dd, J = 13.6, 2.3), 2.80–3.12 (5 H, m), 3. 24–3.38 (1 H, m), 3.48–3.63 (1 H, m), 3.88–4.19 (7 H, m), 6.80 (4 H, m), 7.06–7.20 (4 H, m). ^13^C NMR: 14.2, 15.0, 20.6, 21.5, 22.6, 24.9, 25.5, 28.9, 34.9, 35.5, 38.3, 38.4, 46.8, 48.4, 53.8, 60.7, 66.2, 67.6, 68.9, 80.4, 114.3 (2 C), 114.6 (2 C), 129.3, 129.9 (2 C), 130.3 (2 C), 130.4, 157.6, 157.7, 172.5. Found: m/z 539.3611 [M]+. C_33_H_49_NO_5_. Calculated: m/z 539.3610.

N-(2-(4-(2-(4-((S)-2-carboxy-2-ethoxyethyl)phenoxy)ethyl)phenoxy)ethyl)-2-isopropyl-5-methylcyclohexanaminium chloride (**1f**)

Hydrolysis was carried out using the previously described technique. [5] The material obtained was colorless oil, 0.53 g, with a yield of 77%. ^1^H NMR: 0.82–1.18 (17 H, m), 1.55–1.76 (6 H, m), 2.82–3.08 (5 H, m), 3.36 (1 H, m), 3.51 (1 H, m), 3.93–4. 23 (5 H, m), 4.25–4.51 (2 H, br.m), 6.82–6.87 (4 H, m), 7.15–7.20 (4 H, m). ^13^C NMR: 15.0, 20.8, 21.4, 22.1, 23.9, 25.2, 27.9, 34.5, 34.9, 35.1, 37.9, 45.0, 47.2, 56.3, 63.6, 66.5, 68.7, 79.8, 114.3 (2 C), 114.5 (2 C), 130.1 (3 C), 130.4 (2 C), 131.5, 156.1, 157.6, 174.9. Found: m/z 547.3065 [M]+. C_31_H_46_ClNO_5_. Calculated: m/z 547.3064.

(1R,2S,5R)-2-isopropyl-5-methylcyclohexyl methanesulfonate (**8**)

The material obtained was a yellow oil, with a yield of 95%. The compound was synthesized according to the procedure described earlier in [31].

(1S,2S,4R)-2-azido-1-isopropyl-4-methylcyclohexane (**9**)

The material obtained was a yellow oil, with a yield of 82%. The compound was synthesized according to the procedure described earlier in [32].

(1S,2S,4R)-neomenthylamine (**7**)

The material obtained was colorless crystals, with a yield of 78%. The compound was synthesized according to the procedure described earlier in [33].

### 4.3. Evaluation of Inhibitory Activity against the Main Viral Protease

For assessing the ability to inhibit the main protease (3CLpro), the IC_50_ was the half-maximal inhibitory concentration of the substance at which the fluorescence level reduced by 50% compared to the value obtained without adding the inhibitor [9,10,34]. Fluorescence occurred due to cleavage of the peptide substrate DabcylKTSAVLQ↓SGFRKME(Edans)NH2 by 3CLpro protease. ML188 inhibitor ((R)-N-(4-(tert-Butyl)phenyl)-N-(2-(tert-butylamino)-2-oxo-1-(pyridin-3-yl)ethyl)furan-2-carboxamide, Ambeed Inc, USA) was used as a positive control. In the study, the signal was recorded using the CLARIOstar Plus instrument (BMG Labtech) at 355 and 460 nm for excitation and radiation, respectively, in the kinetic scan mode. Reaction mixtures containing TrisHCl buffer, fluorogenic substrate, 3CLpro, and the compound being tested were prepared and incubated for 30 min in a 384-well plate at 30 °C. The measurement for each compound was carried out in triplicate. The instrument was calibrated using a solution of the peptide that had undergone complete hydrolysis. The accompanying MARS Data Analysis software was used to calculate IC_50_ values.

## 5. Conclusions

Compounds exhibiting an antiprotease activity against the SARS-CoV-2 Mpro were identified among the derivatives of triterpene amides containing (*S*)-ethyl 2-ethoxy-3-(4-hydroxyphenyl) propanoic acid as the pharmacophore fragment. The most active derivatives were the acetylglycyrrhetic and corosolic acid amides. Molecular modeling revealed that binding of the active site of the Mpro to amino acids occurred due to the polar groups and pi-systems of the pharmacophore moiety. The triterpene backbones of these derivatives were able to perform a stabilizing function, holding the compounds on the main protease surface due to hydrophobic interactions. Further chemical modification of glycyrrhetic acid amides seems to be quite relevant for increasing the potential antiprotease activity of compounds belonging to this class against the SARS-CoV-2 Mpro.

## Figures and Tables

**Table 1:** Docking parameters of derivatives (1a-f), according to the IFD protocol, compared to the data obtained for the ML188 non-covalent SARS-CoV-2 Mpro inhibitor (ML188).

Ligand	Docking Parameters, kcal/mol			
	Docking Score	LE	Emodel	IFD Score
1e	−11.784	−0.196	−101.329	−670.17
1c	−10.532	−0.167	−118.702	−667.78
1a	−10.479	−0.178	−104.657	−665.62
1d	−9.843	−0.167	−93.581	−666.84
1b	−8.672	−0.147	−102.7	−666.49
ML188	−8.379	−0.262	−83.299	−666.082
1f	−7.39	−0.2	−86.486	−663.78

**Table 2:** Non-covalent interactions of the new triterpene amides and the ML188 inhibitor with amino acids of the SARS-CoV-2 Mpro active site as a result of molecular docking. The atomic numbering of the new compounds and the ML188 inhibitor are given according to Figure 1 and Figure 2.

Ligand	Interactions	Distance (Å)	Bonding	Bonding Type
1e	Lys137:NZ — 1e:O6’’	4.97	Ionic Bond	Salt Bridge
	Lys137:HZ2 — 1e:O5’’	1.84	Hydrogen Bond	Conventional
	Gln189:HE22 — 1e:O28’	2.15	Hydrogen Bond	Conventional
	1e:H2’ — Thr24:O	1.86	Hydrogen Bond	Conventional
1c	His41:HD1 — 1c:O3’’	1.89	Hydrogen Bond	Conventional
	Asn142:HD22 — 1c:O5’’	1.81	Hydrogen Bond	Conventional
	Gly143:HN — 1c:O6’’	1.91	Hydrogen Bond	Conventional
	Gln189:HE21 — 1c:O11’	2.03	Hydrogen Bond	Conventional
	Gln192:HE22 — 1c:O28’	2.14	Hydrogen Bond	Conventional
	1c:H1’’ — Gln189:OE1	2.16	Hydrogen Bond	Conventional
	His41:Imidazol ring — 1c:Ring A	4.29	Hydrophobic	Pi-Pi Stacked
1a	Asn142:HD22 — 1a:O5’’	1.67	Hydrogen Bond	Conventional
	Gly143:HN — 1a:O6’’	1.67	Hydrogen Bond	Conventional
	Gln189:HE22 — 1a:O28’	1.73	Hydrogen Bond	Conventional
	1a:H1’’ — Glu166:O	1.99	Hydrogen Bond	Conventional
	His41:Imidazol ring — 1c:Ring A	5.49	Hydrophobic	Pi-Pi T-shaped
1d	Lys137:NZ — 1d:O6’’	2.82	Ionic Bond	Salt Bridge
	Lys137:NZ — 1d:Ring B	5.26	Ionic Bond	Pi-Cation
	Thr26:HN — 1d:O3’	2.12	Hydrogen Bond	Conventional
	Glu166:HN — 1d:O28’	2.07	Hydrogen Bond	Conventional
	1d:H3’ — Thr24:O	2.69	Hydrogen Bond	Conventional
1b	1b:H1’’ — Asn142:OD1	2.00	Hydrogen Bond	Conventional
	Lys61:NZ — 1b:O6’’	4.44	Ionic Bond	Salt Bridge
ML188	Asn142:HD22 — ML188:O2	1.71	Hydrogen Bond	Conventional
	His163:HE2 — ML188:N5	2.08	Hydrogen Bond	Conventional
	Glu166:HN — ML188:O3	2.03	Hydrogen Bond	Conventional
	His41:Imidazol ring — ML188:Ring C	5.18	Hydrophobic	Pi-Pi T-shaped
	His41:Imidazol ring — ML188:Ring A	5.27	Hydrophobic	Pi-Pi T-shaped
1f	His41:HD1 — 1f:O3’’	2.04	Hydrogen Bond	Conventional
	Asn142:HD22 — 1f:O6’’	1.67	Hydrogen Bond	Conventional
	Gly143:HN — 1f:O5’’	1.84	Hydrogen Bond	Conventional
	1f:H1’’ — Gln189:OE1	2.42	Hydrogen Bond	Conventional
	1f:H1’’* — Glu166:O	2.35	Hydrogen Bond	Conventional

* Another hydrogen atom of the two at the N1’’+ atom of compound **1f**.

**Table 3:** SARS-CoV-2 Mpro-inhibiting activity of the new triterpene amides in comparison with non-covalent inhibitor ML188.

Ligand	IC_50_, μM
1e	25 ± 3
1c	125 ± 13
1a	100 ± 5
1d	100 ± 5
1b	100 ± 5
ML188	1.6 ± 0.6
1f	<400

## Data Availability

Not applicable.

## Supplementary Materials

The following supporting information can be downloaded at: https://www.mdpi.com/article/10.3390/molecules28010303/s1, Figures S1–S6: NMR spectra for compounds **1f, 5, 6**. Figure S7. Non-covalent interactions of new compounds in the active site of SARS-CoV-2 Mpro compared to the inhibitor ML188.

## Sample Availability

Samples of the compounds may be available from the authors, but only with the permission of the Novosibirsk Institute of Organic Chemistry.
