# Supplementary material for: Triterpenic Acid Amides as Potential Inhibitors of the SARS-CoV-2 Main Protease
Source: Molecules. 2022 Dec 30;28(1):303. doi: 10.3390/molecules28010303 (PMC9822074; doi:10.3390/molecules28010303)
Supplement: Supplementary file 1 [file molecules-28-00303-s001.zip › molecules-2119397-supplementary.pdf]

Supporting Information for:

Triterpenic Acid Amides as Potential Inhibitors of the SARS-CoV-2 Main Protease

Dmitry S. Baev [1,*], Mikhail E. Blokhin [1], Varvara Yu. Chirkova [2], Svetlana V. Belenkaya [3], Olga A. Luzina [1], Olga I. Yarovaya [1], Nariman F. Salakhutdinov [1] and Dmitry N. Shcherbakov [3]

[1] N.N. Vorozhtsov Novosibirsk Institute of Organic Chemistry, 9 Lavrent'iev Avenue, 630090 Novosibirsk, Russia; mblokhin@nioch.nsc.ru (M.E.B.); luzina@nioch.nsc.ru (O.A.L.); ooo@nioch.nsc.ru (O.I.Y.); anvar@nioch.nsc.ru (N.F.S.)

[2] Department of Physical-Chemistry Biology and Biotechnology, Altay State University, 61 Lenina Avenue, 656049 Barnaul, Russia; varvara.chirkova@gmail.com

[3] State Research Center of Virology and Biotechnology VECTOR, Rospotrebnadzor, 630559 Koltsovo, Russia; belenkaya.sveta@gmail.com (S.V.B.); dnshcherbakov@gmail.com (D.N.S.)

* Correspondence: baev@nioch.nsc.ru

INDEX

Figure S1. (S)-ethyl 3-(4-(4-(2-bromoethoxy)phenethoxy)phenyl)-2-ethoxypropanoate ^{1}H NMR spectrum 2

Figure S2. (S)-ethyl 3-(4-(4-(2-bromoethoxy)phenethoxy)phenyl)-2-ethoxypropanoate ^{13}C NMR spectrum 3

Figure S3. (S)-ethyl 2-ethoxy-3-(4-(4-(2-(((1S,2S,5R)-2-isopropyl-5-methylcyclohexyl)amino)ethoxy)phenethoxy) phenyl)propanoate ^{1}H NMR spectrum 4

Figure S4. (S)-ethyl 2-ethoxy-3-(4-(4-(2-(((1S,2S,5R)-2-isopropyl-5-methylcyclohexyl)amino)ethoxy)phenethoxy) phenyl)propanoate ^{13}C NMR spectrum 5

Figure S5. (S)-2-ethoxy-3-(4-(4-(2-(((1S,2S,5R)-2-isopropyl-5-methylcyclohexyl)amino)ethoxy)phenethoxy) phenyl) propanoic acid ^{1}H NMR spectrum 6

Figure S6. (S)-2-ethoxy-3-(4-(4-(2-(((1S,2S,5R)-2-isopropyl-5-methylcyclohexyl)amino)ethoxy)phenethoxy) phenyl) propanoic acid ^{13}C NMR spectrum 7

Figure S7. 2D schemes of non-covalent interactions of compounds in the active site of SARS-CoV-2 Mpro compared to the inhibitor ML188 9

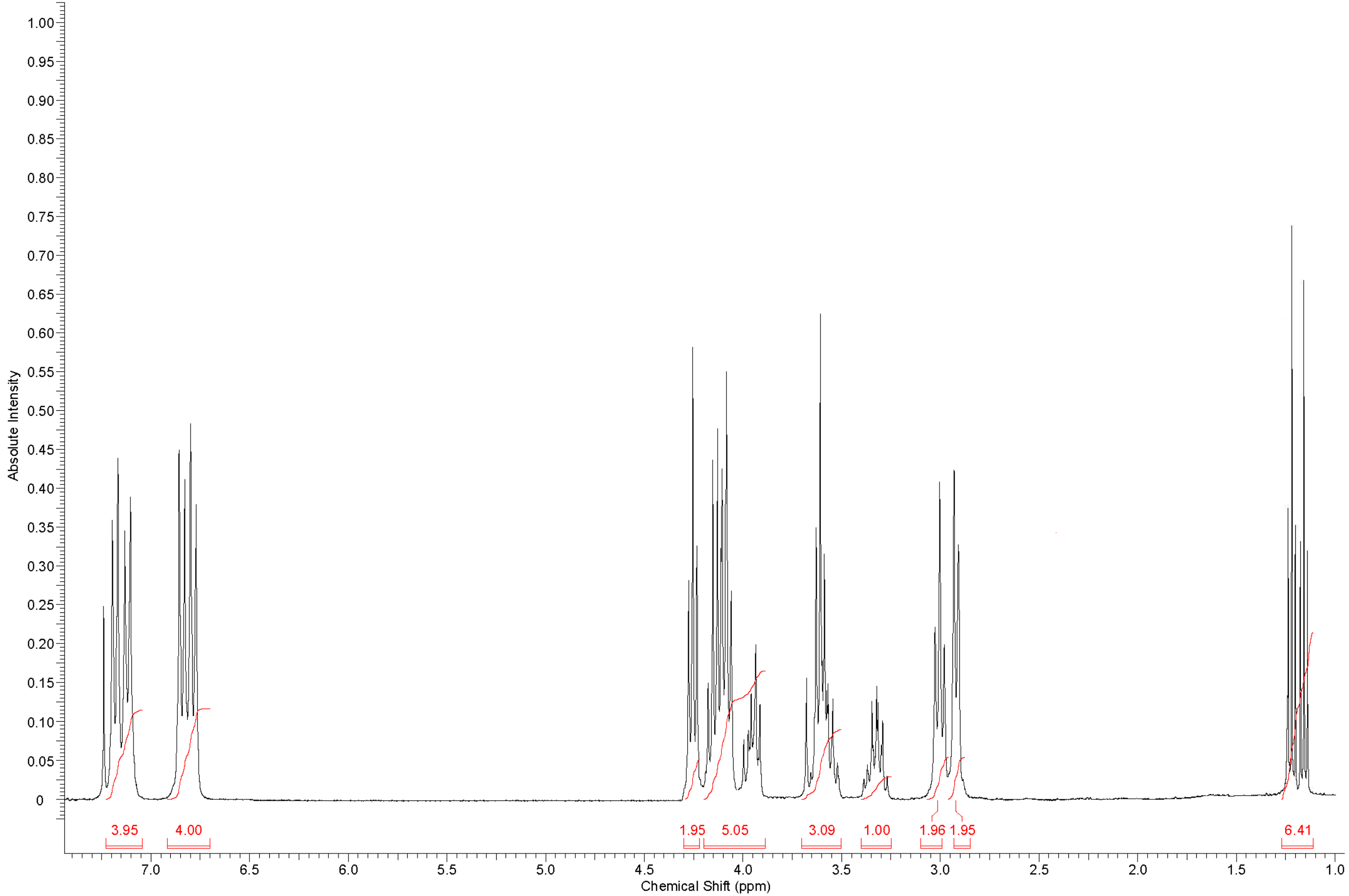

Figure S1. (S)-ethyl 3-(4-(4-(2-bromoethoxy)phenethoxy)phenyl)-2-ethoxypropanoate ^{1}H NMR spectrum.

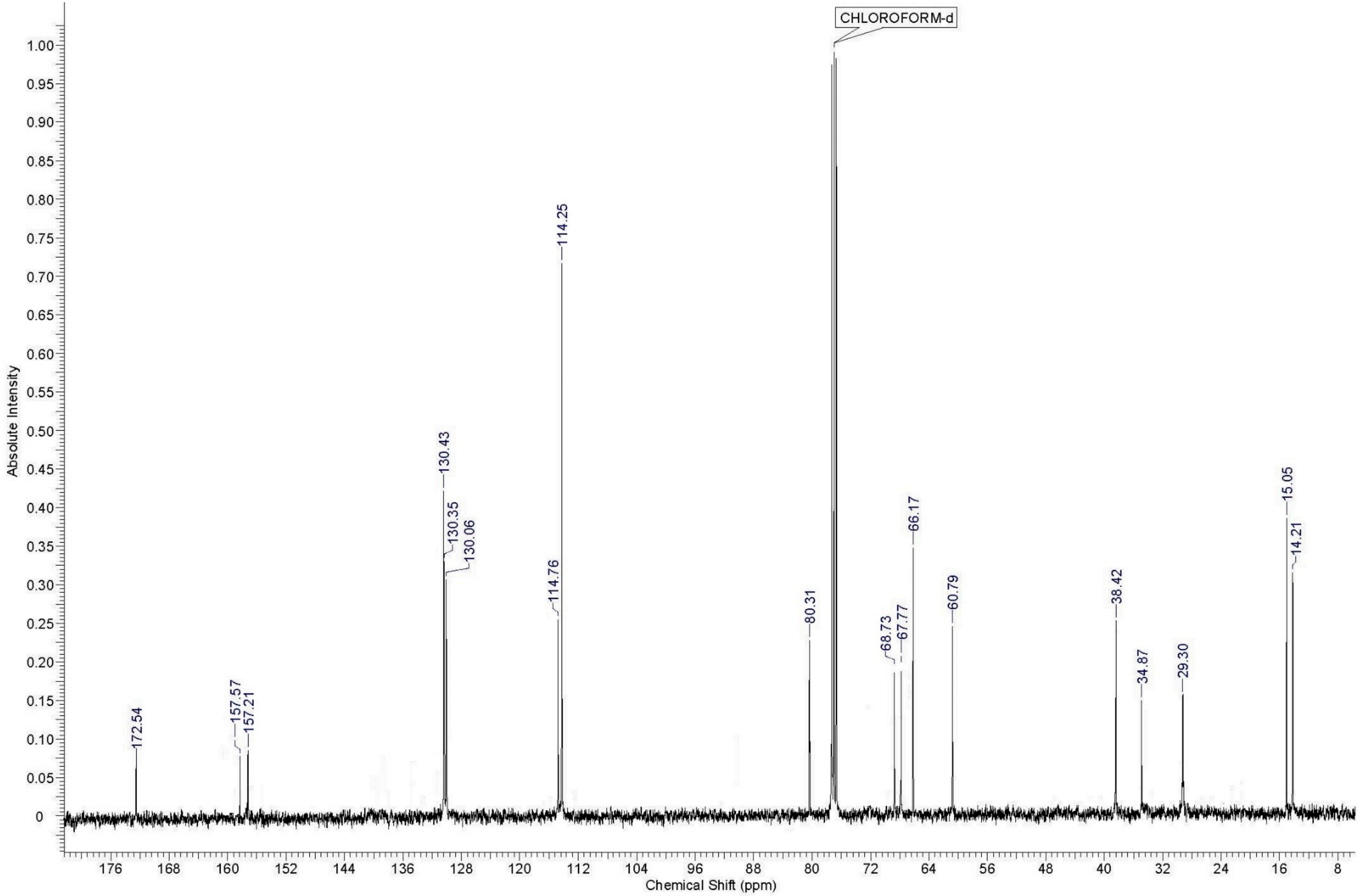

Figure S2. (S)-ethyl 3-(4-(4-(2-bromoethoxy)phenethoxy)phenyl)-2-ethoxypropanoate ^{13}C NMR spectrum.

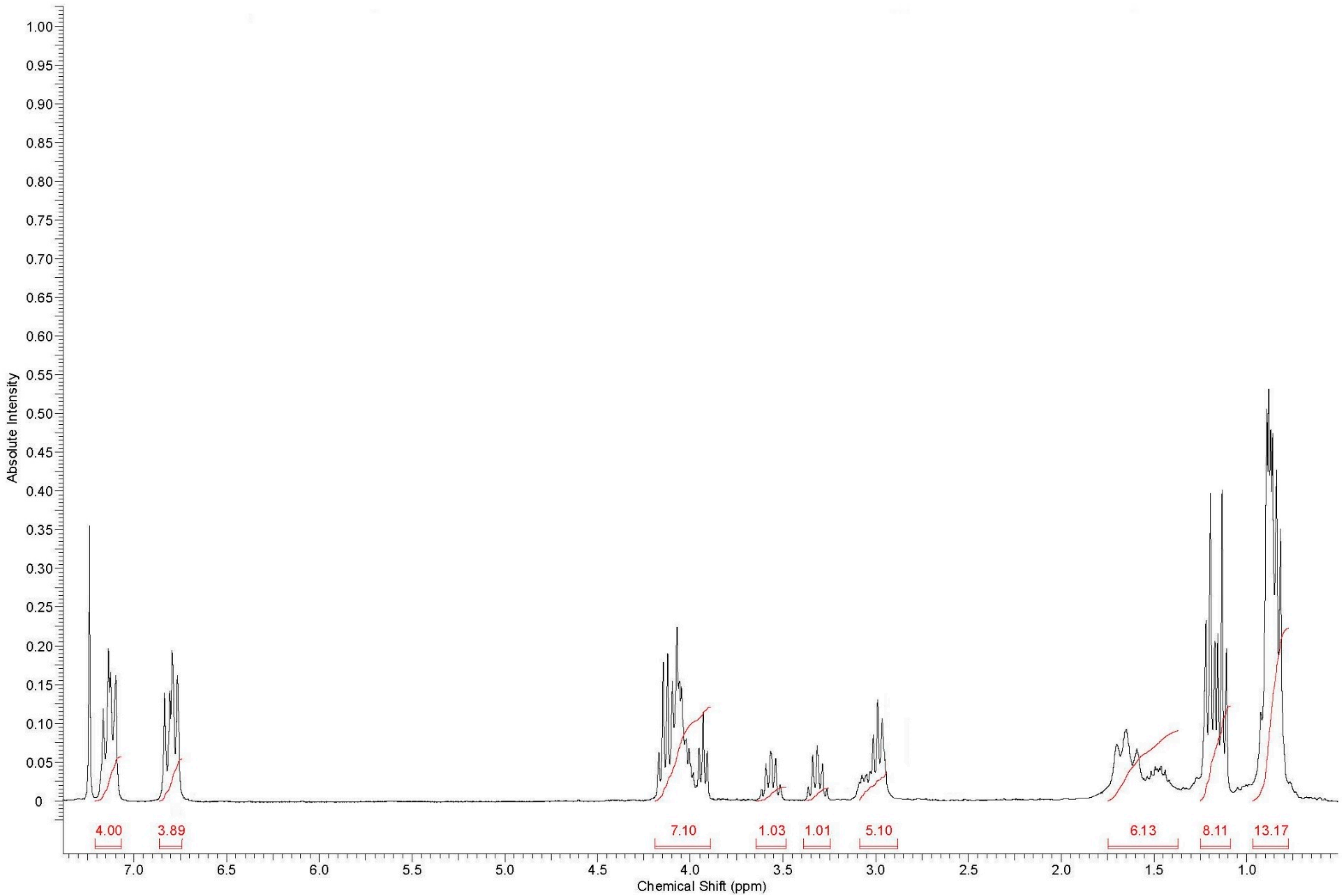

Figure S3. (S)-ethyl 2-ethoxy-3-(4-(4-(2-(((1S,2S,5R)-2-isopropyl-5-methylcyclohexyl)amino)ethoxy)phenethoxy) phenyl)propanoate ^{1}H NMR spectrum.

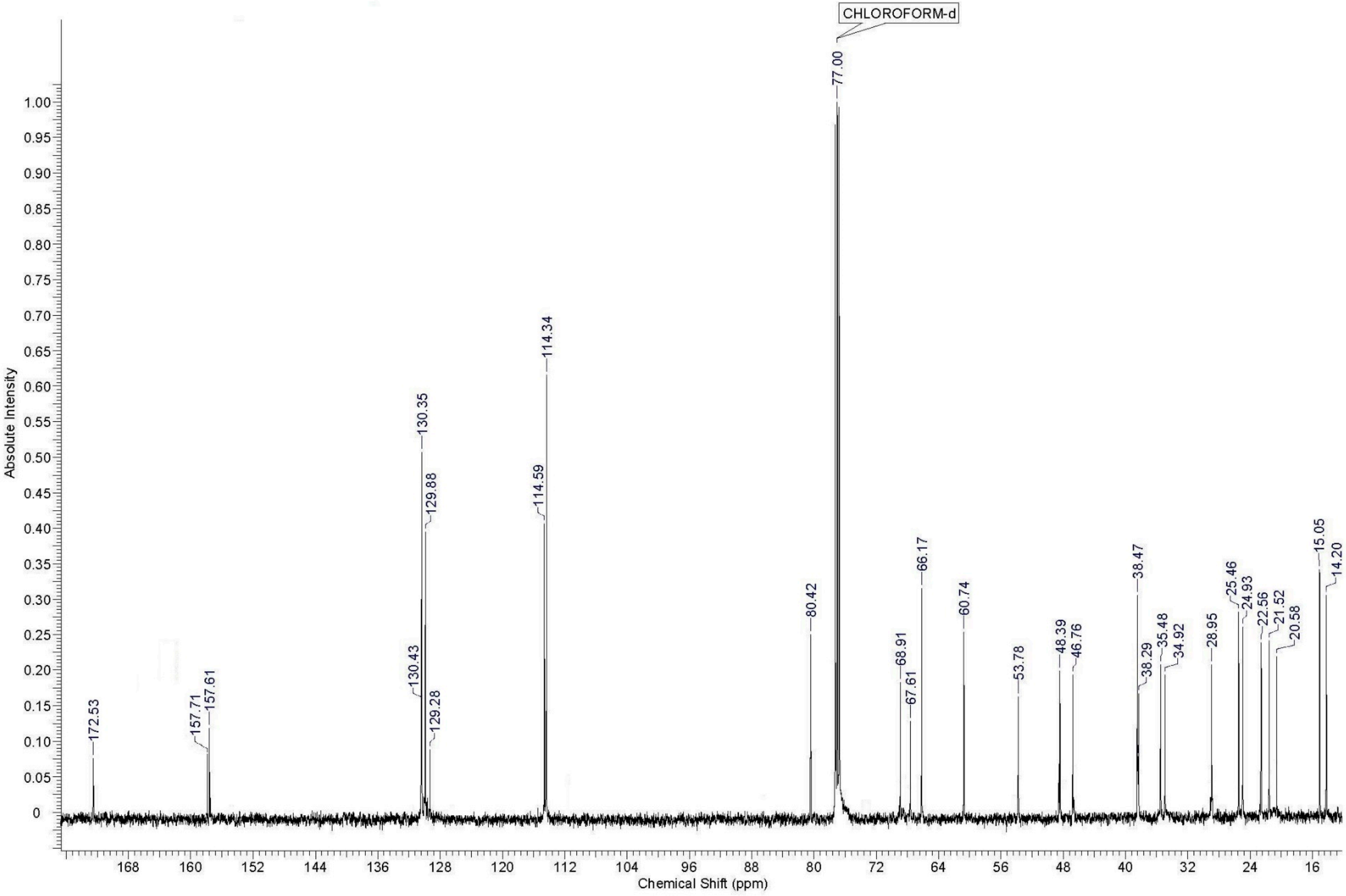

Figure S4. (S)-ethyl 2-ethoxy-3-(4-(4-(2-(((1S,2S,5R)-2-isopropyl-5-methylcyclohexyl)amino)ethoxy)phenethoxy) phenyl)propanoate ^{13}C NMR spectrum.

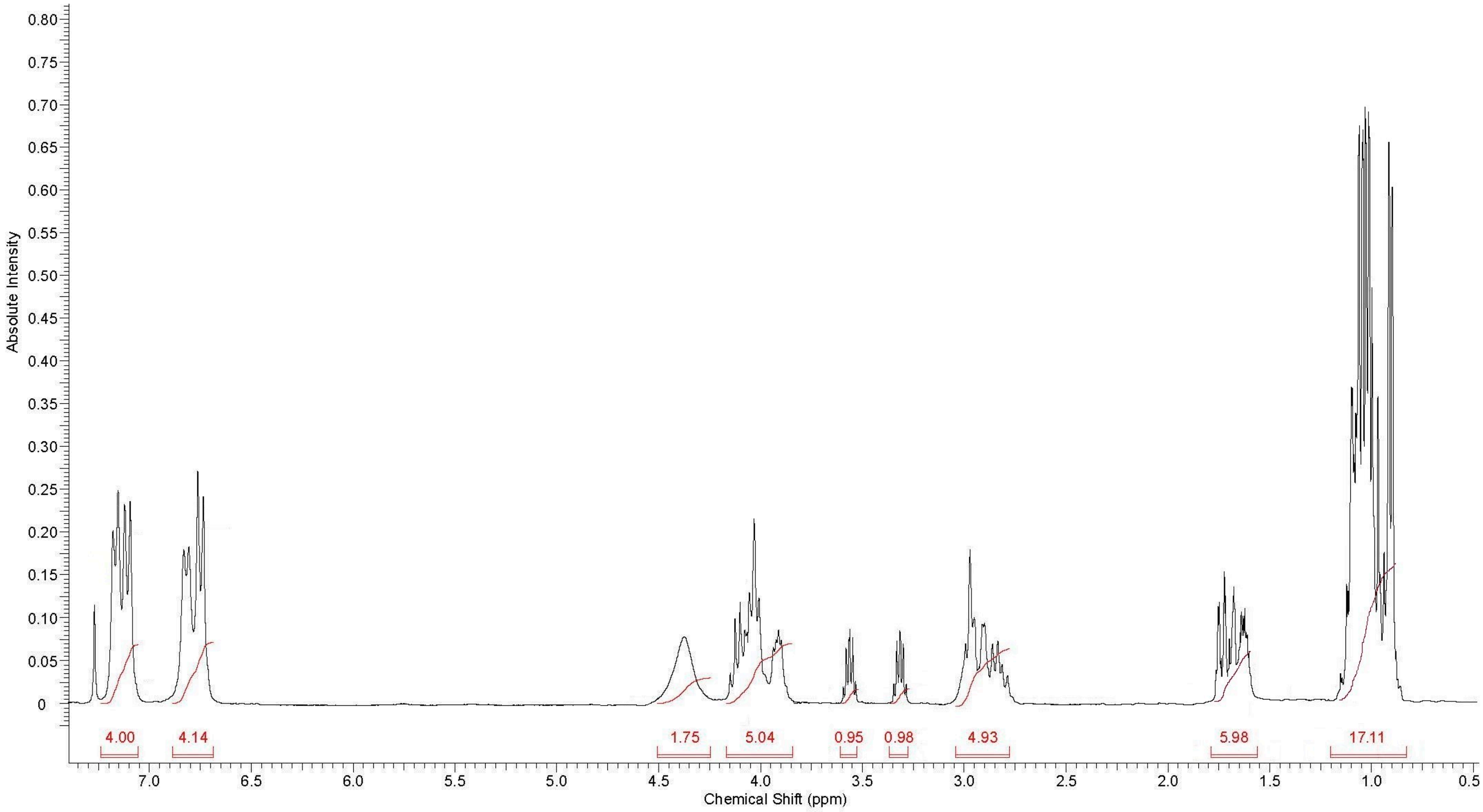

Figure S5. (S)-2-ethoxy-3-(4-(4-(2-(((1S,2S,5R)-2-isopropyl-5-methylcyclohexyl)amino)ethoxy)phenethoxy) phenyl) propanoic acid ^{1}H NMR spectrum.

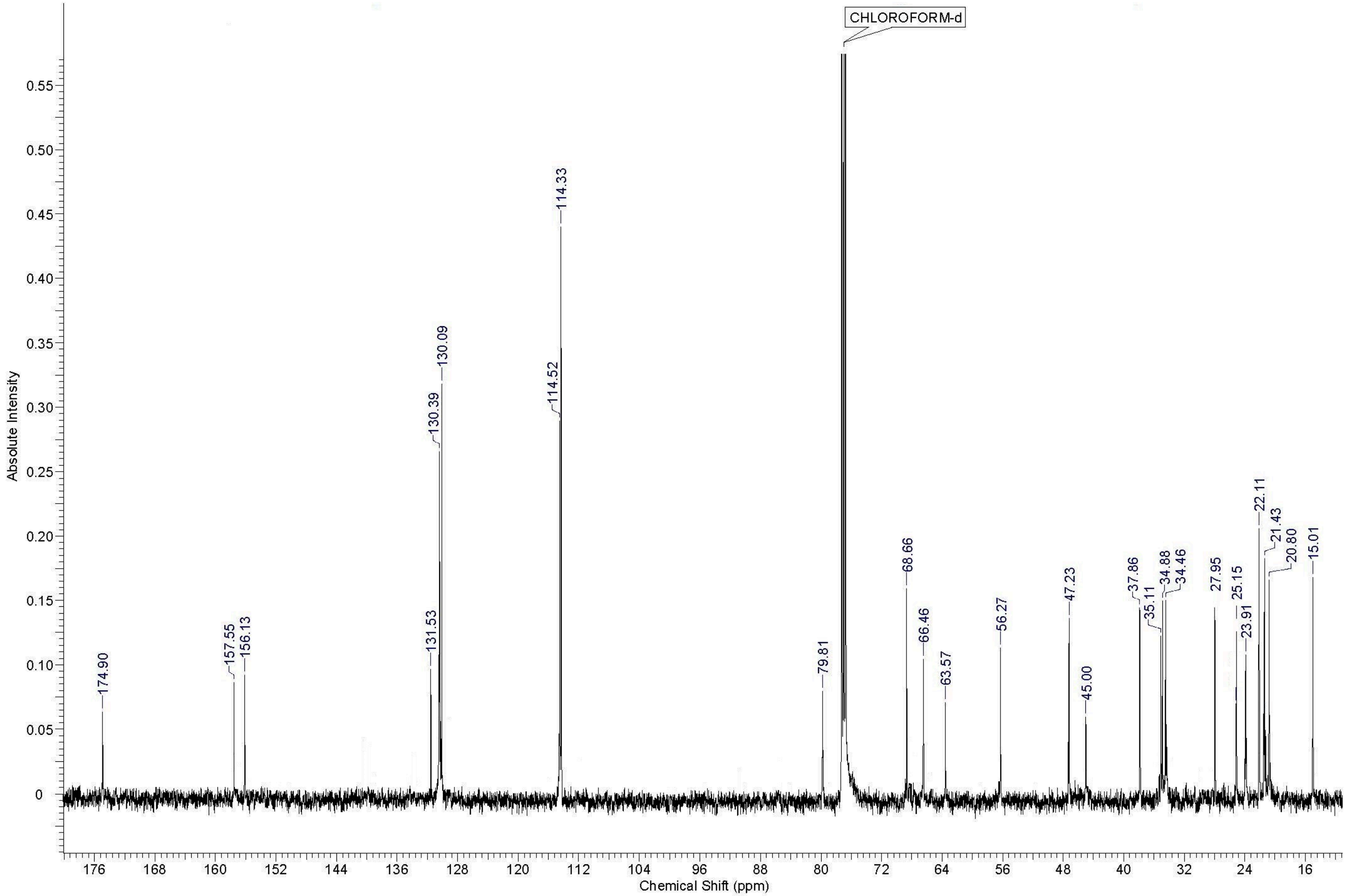

Figure S6. (S)-2-ethoxy-3-(4-(4-(2-(((1S,2S,5R)-2-isopropyl-5-methylcyclohexyl)amino)ethoxy)phenethoxy) phenyl) propanoic acid ^{13}C NMR spectrum.

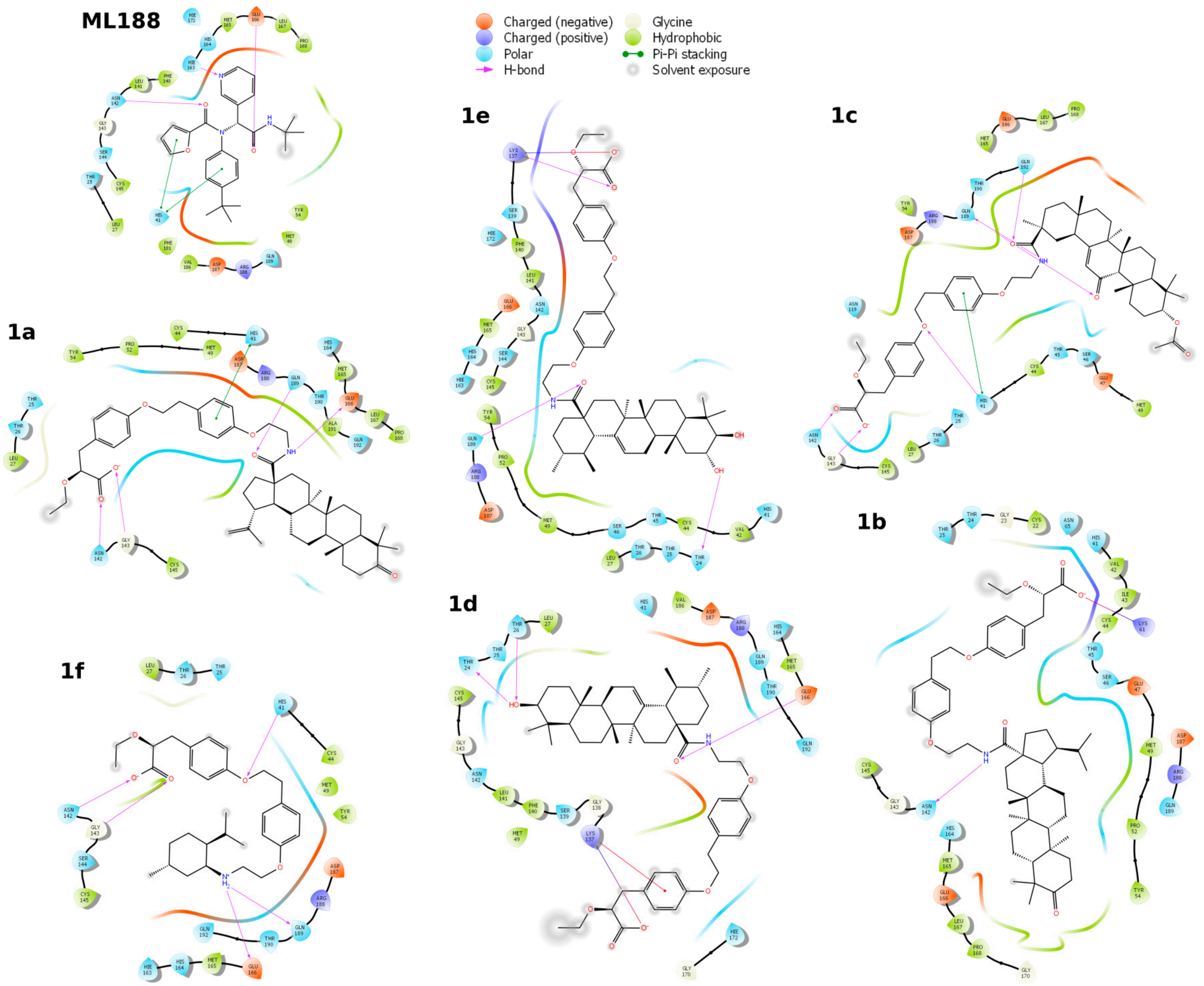

Figure S7. Non-covalent interactions of new compounds in the active site of SARS-CoV-2 Mpro compared to the inhibitor ML188.
